# Use of GenMAPP and MAPPFinder to analyse pathways involved in chickens infected with the protozoan parasite *Eimeria*

**DOI:** 10.1186/1753-6561-3-S4-S7

**Published:** 2009-07-16

**Authors:** Dennis Prickett, Michael Watson

**Affiliations:** 1 Bioinformatics Group, Institute for Animal Health (IAH), Compton, Newbury, RG20 7NN, UK

## Abstract

**Background:**

Microarrays allow genome-wide assays of gene expression. There is a need for user-friendly software to visualise and analyse these data. Analysing microarray data in the context of biological pathways is now common, and several tools exist.

**Results:**

We describe the use of MAPPFinder, a component of GenMAPP to characterise the biological pathways affected in chickens infected with the protozoan parasite *Eimeria. *Several pathways were significantly affected based on the unadjusted p-value, including several immune-system pathways.

**Conclusion:**

GenMAPP/MAPPFinder provides a means to rapidly visualise pathways affected in microarray studies. However, it relies on good genome annotation and having genes reliably linked to pathway objects. We show that GenMAPP/MAPPFinder can produce useful results, and as the annotation of the chicken genome improves, so will the level of information gained.

## Background

Microarrays provide information on the expression of many thousands of transcripts in a single assay. The generation of such data is rapid and increasingly cost-effective. Pathway analysis tools can be used to connect gene expression data from microarrays with existing biological pathways by using specific database identifiers that link reporters with elements in the pathways. This provides a biological context to visualise the results and can be useful for generating testable hypotheses.

There are a number of public sources of pathway information, such as KEGG [1], MetaCyc [2], Reactome [3] and WikiPathways [4]. Software systems that can analyse quantitative data based on these resources include GenMAPP [5], Cytoscape [6], PathVisio [7] and KegArray [8], as well as online tools such as those at Reactome and FatiGO [9].

In this study we applied pathway analysis to identify pathways involved in *Eimeria *infected chickens. *Eimeria *is an obligate intracellular protozoan parasite of chickens of economic importance to the poultry industry [10]. To identify affected pathways we used the software package MAPPFinder [11], a component of the free pathways analysis tool, GenMapp, to identify pathways involved in *Eimeria *infected chickens. This paper is part of a workshop [12] with the aim to present and compare several different methods for the post analysis of microarray data, the results of which have been published in conjunction with this paper [13].

## Results

From 20,465 unique probes on the array, 12,038 unique Ensembl genes were identified. Of those, 1175 genes (9.8%) could be mapped to the 85 inferred chicken pathways available through GenMAPP.

**Figure 1:**
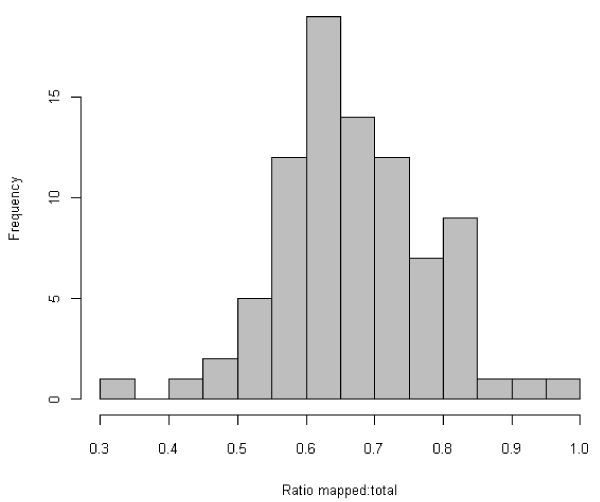
**Distribution of pathway coverage in GenMAPP**. The histogram shows the log2 of the ratio of number of genes mapped to total number of genes on the pathway. The majority of pathways have a value greater than 0.5, which represents a figure of 50% of genes in the pathway are mapped to probes on the array.

The distribution of the ratio of genes mapped to total genes in the pathway is shown in Figure 1 and has a mean of 0.67. This shows that in most cases the majority of the entities in a pathway are represented in the data by at least one gene.

**Figure 2:**
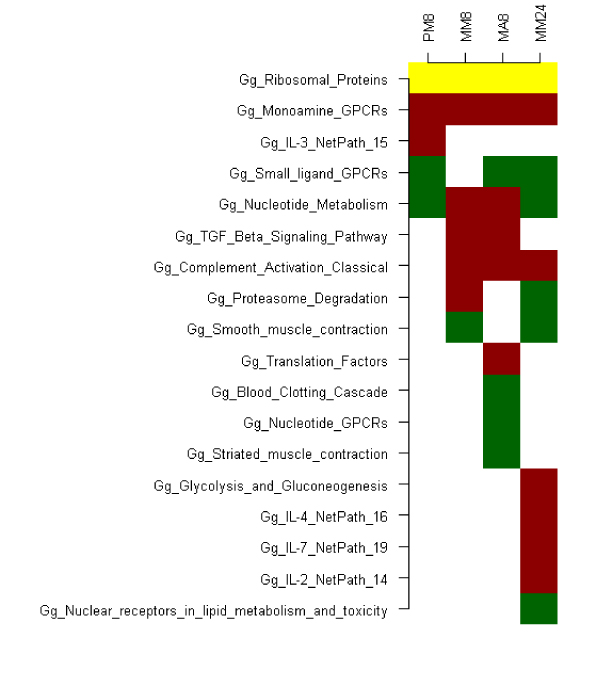
**Up- and down- regulated pathways**. Heatmap of up- and down- regulated pathways using an unadjusted p-value cut-off of p <= 0.05. Red signifies up-regulation, green down-regulation and yellow indicates the pathway was both up- and down- regulated. White indicates no significance for that pathway in that experiment.

Using an adjusted p-value cut-off of 0.05, two pathways, "ribosomal proteins" (PM8 and MA8) and small ligand GPCRs (PM8), were significantly affected. Using an unadjusted p-value cut-off of 0.05, 18 pathways were significantly affected in one or more experiments (Figure 2).

Monoamine GPCRs appear consistently upregulated and small-ligand GPCRs consistently downregulated. The IL2, IL4 and IL7 pathways are upregulated in MM24. The IL3 pathway is upregulated in PM8 but is not so during subsequent infections. The TGF beta signalling pathways is upregulated in MM8 and MA8, but not PA8 nor MM24. The ribosomal proteins pathway is both up- and down- regulated in all experiments.

## Discussion

We were able to visualise the effects of an *Eimeria *infection on chickens using the pathway analysis tool MAPPFinder. Using the adjusted p value the results of the pathway analysis were inconclusive, however using the unadjusted p value we were able to note effects in several different pathways. The biological interpretation of these results were compared with other methods and are presented in a summary paper published in conjunction with this paper [13].

We included data using unadjusted p-values as the lack of significant results using the adjusted p-values results from a lack of power when performing the statistical test for over-representation. For example, when looking at the Ribosomal Proteins pathway, there are 56 objects in the pathway, with 42 being represented by probes on our array. However, at PM8, only 8 of those genes matched the criteria. This was typical throughout the results, with only a small fraction of the genes in the pathway fitting the criteria, thus hugely reducing the power of the analysis. It is possible that we must conclude from this that nothing of significance is occurring in our experiment, however, this seems unlikely given that it is well known that *Eimeria *induces an immune response in chickens. In the light of this knowledge, the weak results from the analyses would appear to be due to a poor mapping of genes to pathways combined with the problem that we are measuring transcription. An increase or decrease in transcription is only one way in which a cell can activate certain pathways, with others being post-transcriptional regulation of transcripts or proteins, post-translational modification of proteins, and biochemical activation of proteins, none of which are measured by microarrays.

The quality of pathway analysis could be increased by improvements in the quality of annotation of the chicken genome, in microarray design, and in the pathways used for analysis. Pathway analysis was based on less than 10% of the genes that could be assigned an Ensembl gene id. The microarray used in this study was designed in 2005 and since then there has been an additional chicken genome assembly and 20 additional versions of the Ensembl database. Microarrays based on current assemblies may increase the coverage of the pathways. Additionally the inferred pathways for chickens have fewer genes represented on the pathways than human GenMapp pathways. Improvements in the pathway mapps would also improve the quality of pathway analysis.

## Conclusion

In this study we were able to identify several pathways affected in chickens infected with the protozoan parasite *Eimeria *using GenMapp/MAPPFinder. This demonstrates that this pathway analysis tool could be useful for microarray experiments using chickens. Further improvements in annotation, microarray design, and pathway design would help to enhance the ability to use pathway analysis to understand biological processes.

## Materials and methods

### Microarray dataset

The microarray used in this study was the Arkgenomics chicken 20 K oligo microarray [14] which consists of 20,460 probes annotated using the IMAD system [15]. This system uses NCBI BLAST to match microarray probes to Ensembl transcripts, from which Ensembl gene ids were derived.

### Experiment

Two-week-old chickens infected with *Eimeria maxima *were challenged two weeks later either with *Eimeria maxima *(MM), *Eimeria acervulina *(MA), or PBS (PM). Samples were taken for analysis at 8 hours (MM8, MA8, and PM8) and 24 hours (MM24) after the challenge. This allows us to analyse the differences between a primary and secondary response (MM8/PM8), differences due to a challenge with a different species (MM8/MA8), and changes over time (MM8/MM24).

### MAPPFinder Analysis

For the characterisation of the biological processes affected in this study, we used GenMAPP 2.1 and MAPPFinder 2.1 to produce lists of significantly regulated pathway [5,11,16]. For the analysis of chicken microarray data, GenMAPP has 85 inferred mapps based on human pathways. Values used in this study were normalised log-ratios obtained by lowess normalisation. MappFinder analysis of these datasets used two criteria, either an increase (=> 1.5) or a decrease <= 1.5) in gene expression to generate lists of significantly affected pathways. MappFinder analysis provides two p values, one using the Westfall-Young adjustment calculation for the family-wise error rate for multiple testing and the unadjusted p value [11]. A pathway was defined as significantly affected if the p value <= 0.05

## Competing interests

The authors declare that they have no competing interests.

## Authors' contributions

DP carried out the analysis and assisted in the writing of the paper. MW advised on the analysis and assisted in the writing of this paper.

## References

[B1] Kanehisa M (2008). KEGG for linking genomes to life and the environment. Nucleic Acids Res.

[B2] Caspi R (2008). The MetaCyc Database of metabolic pathways and enzymes and the BioCyc collection of Pathway/Genome Databases. Nucleic Acids Res.

[B3] Matthews L (2009). Reactome knowledgebase of human biological pathways and processes. Nucleic Acids Res.

[B4] Pico AR (2008). WikiPathways: pathway editing for the people. PLoS Biol.

[B5] Salomonis N (2007). GenMAPP 2: new features and resources for pathway analysis. BMC Bioinformatics.

[B6] Yeung N (2008). Exploring biological networks with Cytoscape software. Curr Protoc Bioinformatics.

[B7] van Iersel M (2008). Presenting and exploring biological pathways with PathVisio. BMC Bioinformatics.

[B8] Okuda S (2008). KEGG Atlas mapping for global analysis of metabolic pathways. Nucleic Acids Res.

[B9] Al-Shahrour F, Diaz-Uriarte R, Dopazo J (2004). FatiGO: a web tool for finding significant associations of Gene Ontology terms with groups of genes. Bioinformatics.

[B10] Williams RB (2006). Tracing the emergence of drug-resistance in coccidia (Eimeria spp.) of commercial broiler flocks medicated with decoquinate for the first time in the United Kingdom. Vet Parasitol.

[B11] Doniger SW (2003). MAPPFinder: using Gene Ontology and GenMAPP to create a global gene-expression profile from microarray data. Genome Biol.

[B12] EADGENE Annotation Workshop. http://www.eadgene.info/NewsandEvents/EADGENEEvents/EADGENEandSABREPostanalysesWorkshop/AnnotationWorkshopResults/tabid/345/Default.aspx.

[B13] Hedegaard J (2009). Methods for interpreting lists of affected genes obtained in a DNA microarray experiment. BMC Proceedings.

[B14] Arkgenomics. http://www.arkgenomics.org/index.php.

[B15] Prickett MD, Watson M (2009). IMAD: flexible annotation of microarray sequences. BMC Proceedings.

[B16] GenMAPP. http://www.genmapp.org.

